# A Puzzling Thank You #2

**DOI:** 10.3928/24748307-20231213-01

**Published:** 2024-01

**Authors:** Michael K. Paasche-Orlow, Sam Barocas

Thank you to all the reviewers listed on the next page for all of your efforts this past year; the 10 highest-rated reviewers are identified with an asterisk.

Please submit complete puzzles (**Figure 1**) via email to mpo@tufts.edu. Be in the top five and we will make a donation in your honor to an adult literacy program.

**Figure 1.**
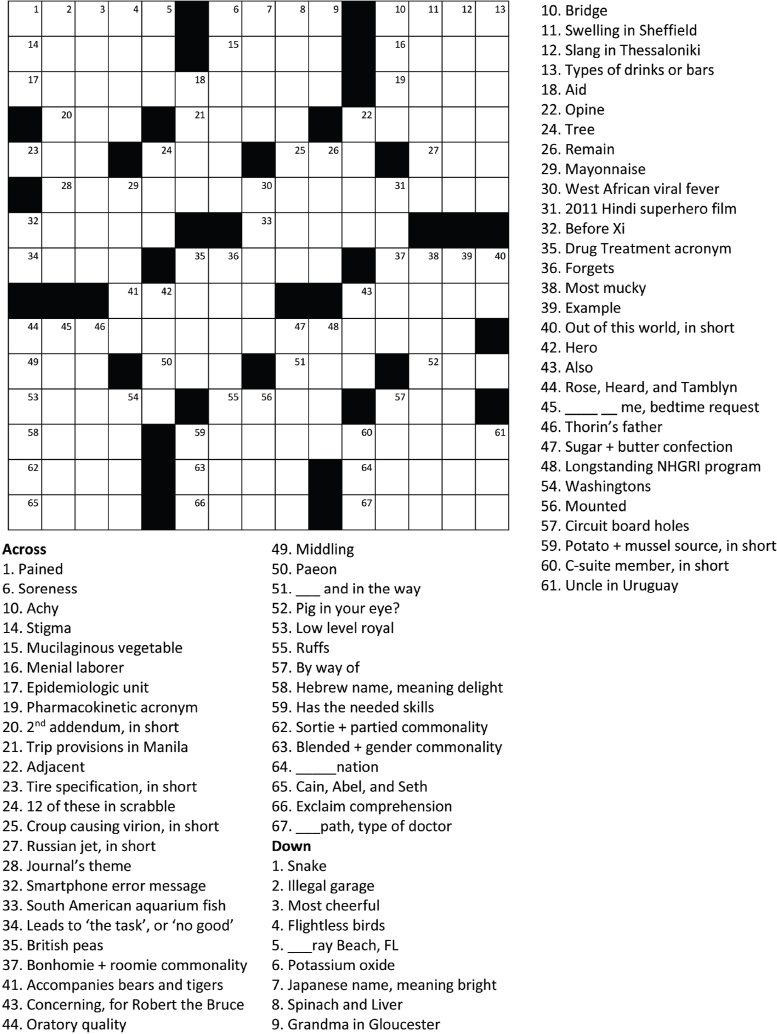
HLRP Puzzle 2024.

## HLRP: Health Literacy Research and Practice Reviewers 2023

Traci Alberti
Kathryn Anderson
Evans Ansu-Yeboah*
Stacy Cooper Bailey*
Howard Cabral
Elena Carbone
Flaviane Cesar
Alex Chow
Karen Colorafi*
Tahereh Dehdari
Radhika Devraj
Anthony Dissen
Iris Feinberg
Sasha Fleary
Mirjam Fransen
Suad Ghaddar
James Griffith
Eleanor Hall
Carol Howe
Emily Hurstak*
Jennifer Innis
Elizabeth LeQuieu*
Mark Macek
Jennifer Manganello
Cathy D. Meade
Melanie Messer
Ann Miller
Don Nutbeam
Raymond L. Ownby
Rebecca Pille
Kathleen Porter
Patricia Quinlan
Doris Ravotas
Donald Rubin*
Katie Shradley*
Reshmi Singh
Paul Smith
Carl Streed
Jessica Thompson*
Laura Wagner
Barry D. Weiss*
Richard White*

